# Hot lamination and origami assembly fabrication of miniaturized compliant mechanism

**DOI:** 10.1016/j.mex.2021.101458

**Published:** 2021-07-17

**Authors:** Takashi Ozaki, Norikazu Ohta, Motohiro Fujiyoshi

**Affiliations:** Toyota Central R&D Labs. Inc., Aichi, Japan

**Keywords:** Compliant mechanism, Hot lamination, Origami, Micro robotics

## Abstract

We developed a process for fabricating a miniaturized hinged mechanism for microrobots by customizing our previously reported flapping-wing method to fabricate microscopic wing structures resembling insects. Flexible hinges were realized by removing the titanium layers of the structure, which were previously sandwiched between thin polyimide layers. A three-dimensional structure can also be realized via origami-like assembly owing to the hinge structure, where the hinge is made to work as a crease. Details of our method and a reference design are reported through a demonstration of a translation-to-rotation conversion mechanism for a piezoelectric actuator.

• Simple fabrication using a single hot lamination and origami-like assembly.

• Method based on FPC manufacturing technologies would be easy to implement in a variety of research.

• Transmission mechanism for piezoelectric actuators was demonstrated, verifying that the structures fabricated by this process function appropriately.

## Graphical abstract

**Figure:**
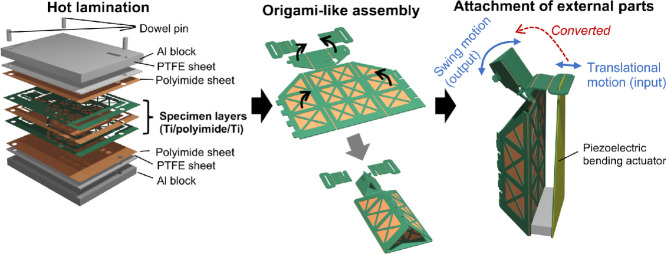

Specifications Table

**Table:** 

Subject Area:	Engineering
More specific subject area:	*Mechanical engineering*
Method name:	*Hot lamination and origami assembly of miniaturized compliant mechanism*
Name and reference of original method:	*Takashi Ozaki, Kanae Hamaguchi, “Batch fabrication process of biomimetic wing with high flexibility of stiffness design for flapping-wing micro aerial vehicles,” MethodsX, Volume 7, 2020,* https://doi.org/10.1016/j.mex.2020.101121.
Resource availability:	*The resource that support the findings of this study are available from the corresponding author upon reasonable request.*

## Background

In recent years, research on insect-scale microrobots has attracted much attention. Various types of robots, such as walking robots [1], swimming robots [2], and flying vehicles [3], have been studied. At very small scales, a significant amount of energy is lost due to friction in rotating mechanisms. Therefore, a compliant mechanism using the elastic deformation of a structure as a movable joint is a good choice for small mechanisms in microrobots. However, fabricating such small and complex three-dimensional mechanisms is difficult, and appropriate techniques are yet to be established. For example, a research group at Nihon University reported a millimeter-scale walking robot actuated by a shape-memory alloy [4]. Their transmission mechanism was composed of manually-assembled silicon parts. Such an assembly is difficult because it requires a large amount of time and effort and depends on the dexterity of the individual. Another approach is a layer-by-layer process based on the semiconductor microfabrication technology. By layering a flexible layer such as photosensitive polyimide on a pattern of a rigid material such as silicon or any metal, a planar compliant mechanism can be fabricated. For example, Zotov et al. fabricated a hinge made of photosensitive polyimide on a silicon-on-insulator wafer and assembled it similar to origami for realizing a three-dimensionally arranged inertial sensor unit [5]. Matei et al. and Asamura et al. also reported miniatured actuators with compliance mechanism using similar processes [6,7]. The semiconductor microfabrication process enables the formation of various structures on a wafer of a fixed area at once. Smaller the device size, the more structures can be fabricated at once, thus lowering the cost. However, when the device size becomes several tens of millimeters (insect scale), the number of structures that can be fabricated on a wafer becomes small, making the semiconductor fabrication unsuitable. One of the most successful micro-robotics studies was a Harvard University project [8], in which a transmission mechanism for a flapping-wing robot was developed by laminating carbon fiber-reinforced plastic (CFRP) and polyimide sheets. The mechanism were realized using polyimide layer. Three-dimensional structures were created by folding the sheets at the hinges, realizing an origami-like assembly. Their mechanism consists of a minimum of five layers of lamination; a polyimide layer is sandwiched between two CFRP layers, and adhesive layers are placed between the polyimide and CFRP layers. All layers are patterned by ultraviolet (UV) laser cutting. Then, they are heat-pressed by vacuum-packed.hot pressing.

In this paper, we report a new versatile method for fabricating micro-compliant mechanisms, whose structural concept was inspired by an earlier report of the Harvard University group [8]; our structure was also created using a lamination process and polyimide hinges. This proposed method is based on our previously reported hot lamination method for fabricating microscopic wing structures resembling insects [9], which has major advantages such as simplicity and accessibility—the process consists of a single hot lamination and origami-like assembly. We employed metal as the rigid layer and an adhesive-less thermal bondable polyimide product as the flexible hinge layer. This configuration reduced the number of layers required to only three. CFRP, employed in the previous research [8], has the advantage of very high specific strength. However, it has some disadvantages such as fraying of fibers on the cut surface, low heat resistance, and high material cost. Thickness availability is also limited; few products are available in thicknesses below 100 um. Another possible disadvantage is that special processes such as vacuum-packed heat presses for curing and bonding may not be accessible to researchers. In contrast, metal sheets are inexpensive, have smooth edges if wet etched, and are commonly available in thicknesses ranging from very high to as low as 10 um, allowing for greater design flexibility. In addition, our method can be realized using a combination of existing (off-the-shelf) technologies and low-cost instruments. As a result, the proposed method may potentially be used by researchers in various institutes. In this paper, we introduce the fabrication process in detail, demonstrating its use by fabricating a transmission mechanism for a piezoelectric actuator as an example.

## Structure of proposed mechanism

**Fig. 1:**
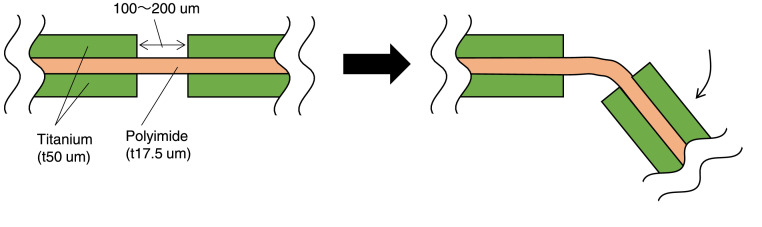
Concept of compliant hinge structure fabricated using proposed method.

Fig. 1 shows a cross-sectional illustration of a basic structure, which consists of a polyimide layer sandwiched between two titanium layers, fabricated using our proposed process. The hinge is realized by removing the titanium layer, leaving only a thin polyimide layer. In typical experiments, the thicknesses of the titanium and polyimide layers were 50 and 12.5 µm, respectively; however, we confirmed that other thicknesses also worked well. For the polyimide layer, we used a product with an adhesive-less thermal bondable product used in flexible printing circuit (FPC) manufacturing, UPILEX-VT (Ube Industries, Ltd., Tokyo, Japan). We expect that the titanium layer can also be replaced with other metallic materials. The hinge can be used not only as a movable joint but also as a crease; the structure can be assembled like origami, by folding the rigid layer along the hinges to form a three-dimensional structure.

**Fig. 2:**
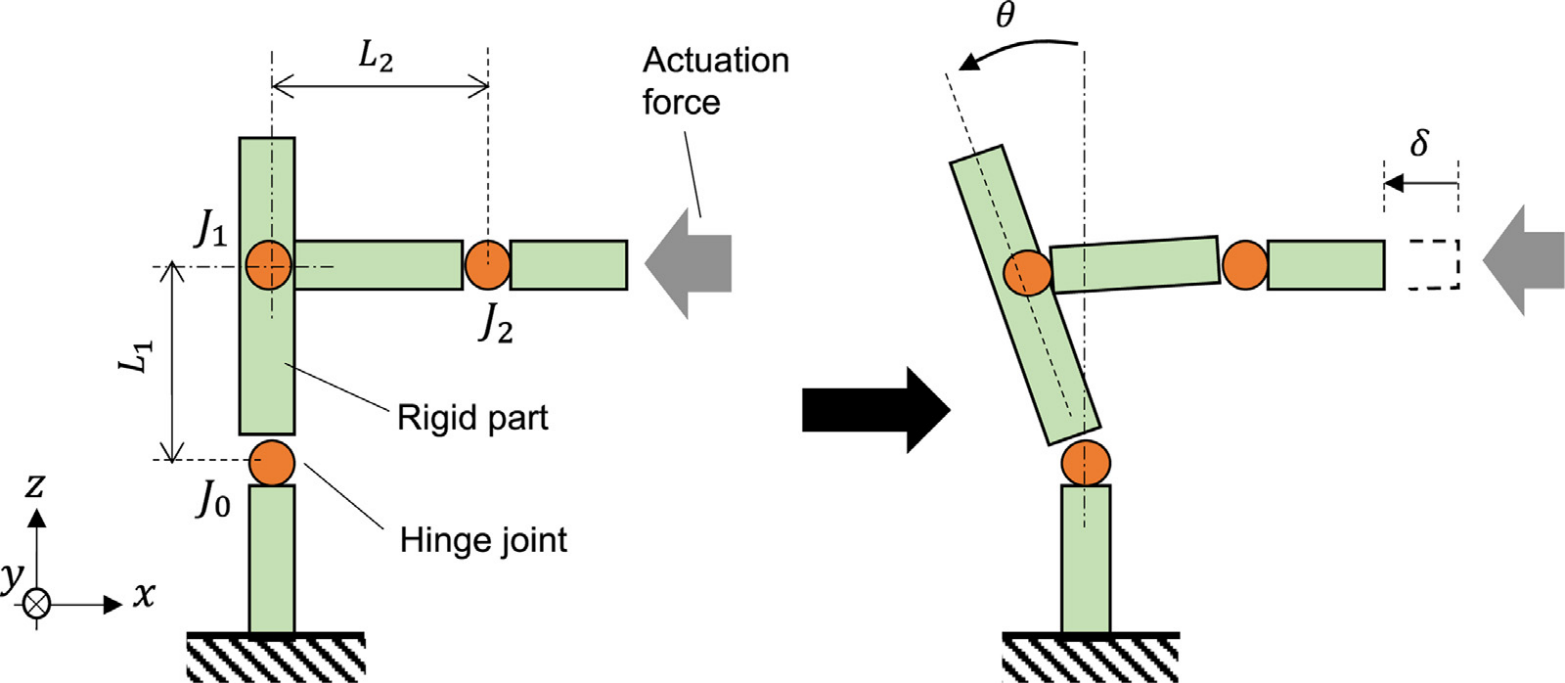
Concept of demonstration mechanism.

**Fig. 3:**
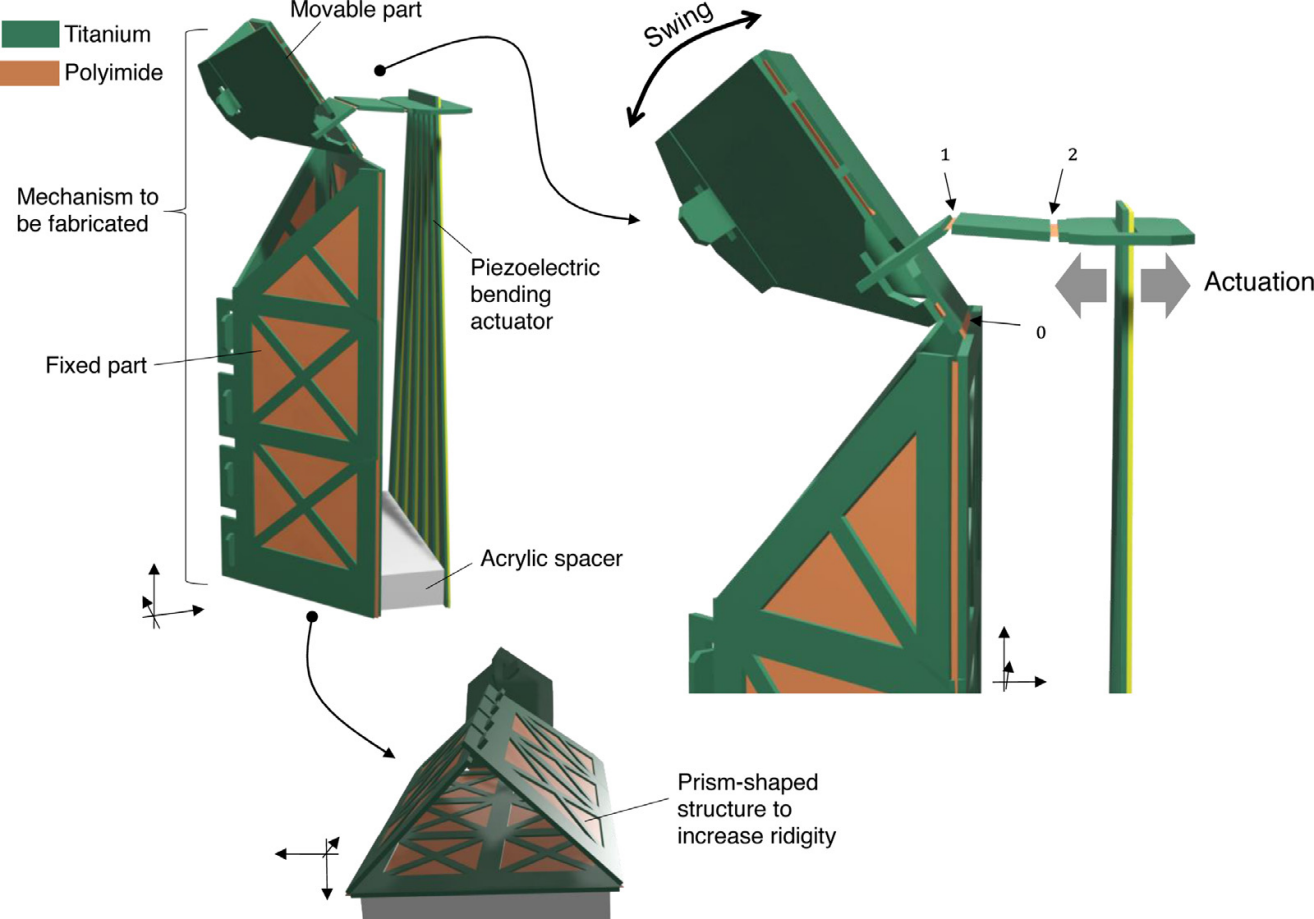
Fabricated mechanism.

In this study, we demonstrated our fabrication technique by developing a transmission mechanism, as described before. Fig. 2 shows the conceptual transmission mechanism, which uses three hinges $:\;J_{0},\;J_{1}$, and $J_{2}$. This is a type of translation-rotation conversion mechanism; by pushing one end with an external actuator, a part of the mechanism rotates. A piezoelectric bending actuator was used. In this demonstration, we designed $L_{1}$ and $L_{2}$ to be 2.0 mm in length. The output rotational angle θ can be calculated as $\theta =\arcsin(\delta /L_{1})$ if δ is sufficiently small relative to $L_{1}$. Fig. 3 shows a concrete image of the fabricated mechanism. The upper part of the mechanism is movable and rotated by the actuation force from the transmission mechanism. A close-up of the transmission mechanism is shown on the right side of Fig. 3. Here, joints $J_{0},\;J_{1},\;\text{and}\;J_{2}\;$are identical to those in Fig. 2. For an ideal motion, the fixed and movable parts should be sufficiently rigid relative to the hinges. The fixed part is shaped like a prism to increase its rigidity, as shown at the bottom of Fig. 3, which is realized using an origami-like folding assembly process.

## Fabrication process

First, the patterned titanium and polyimide layers were prepared using wet etching and laser cutting, respectively. Wet etching of metals is commonly used to create integrated circuit lead frames or other metal components, which is used by many manufacturers. We manufactured the titanium sheets of this study at Hirai Seimitsu Kogyo Co. Ltd. (Osaka, Japan). The laser cutting of polyimide, which was performed at Nano-Process Co. Ltd. (Hamamatsu, Japan), is also widely used as a manufacturing technology for FPCs. Both processes utilize technologies that have been put into practical use. As a result, this step can be carried out by many manufacturing companies, research institutes, etc. without the need for novel technology developments and should therefore be accessible to many researchers. The designs of the titanium and polyimide layers are shown in Fig. 3. Holes in the outer frames of both layers were used for alignment. Inside the outer frame, the specimen shapes were suspended via narrow bridges of width = 200 µm.

**Fig. 4:**
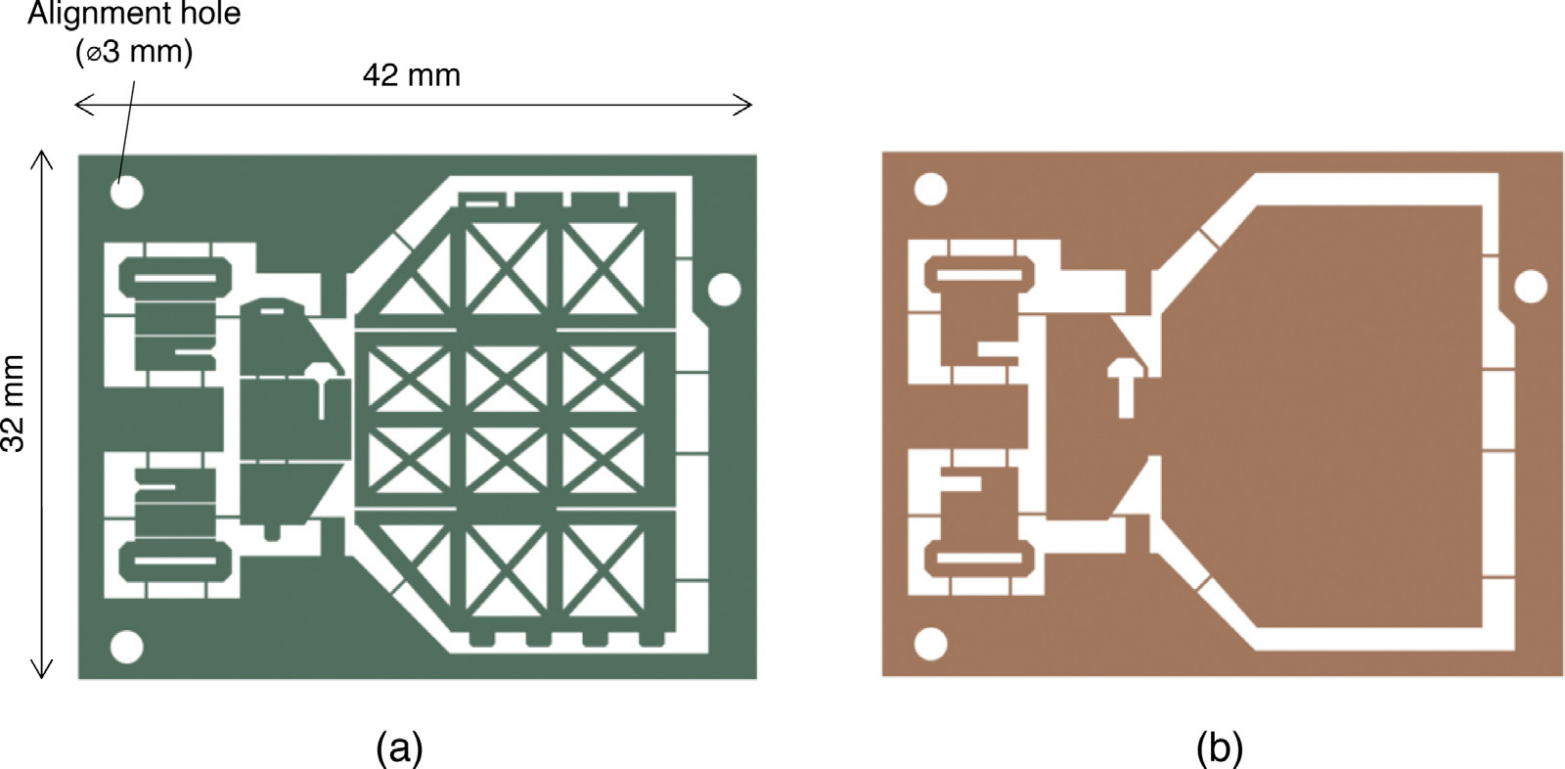
Designs of (a) rigid titanium layer and (b) flexible polyimide layer (raw DXF files are attached as supplementary data).

The steps of the newly developed fabrication process are shown in Fig. 4.

**Fig. 5:**
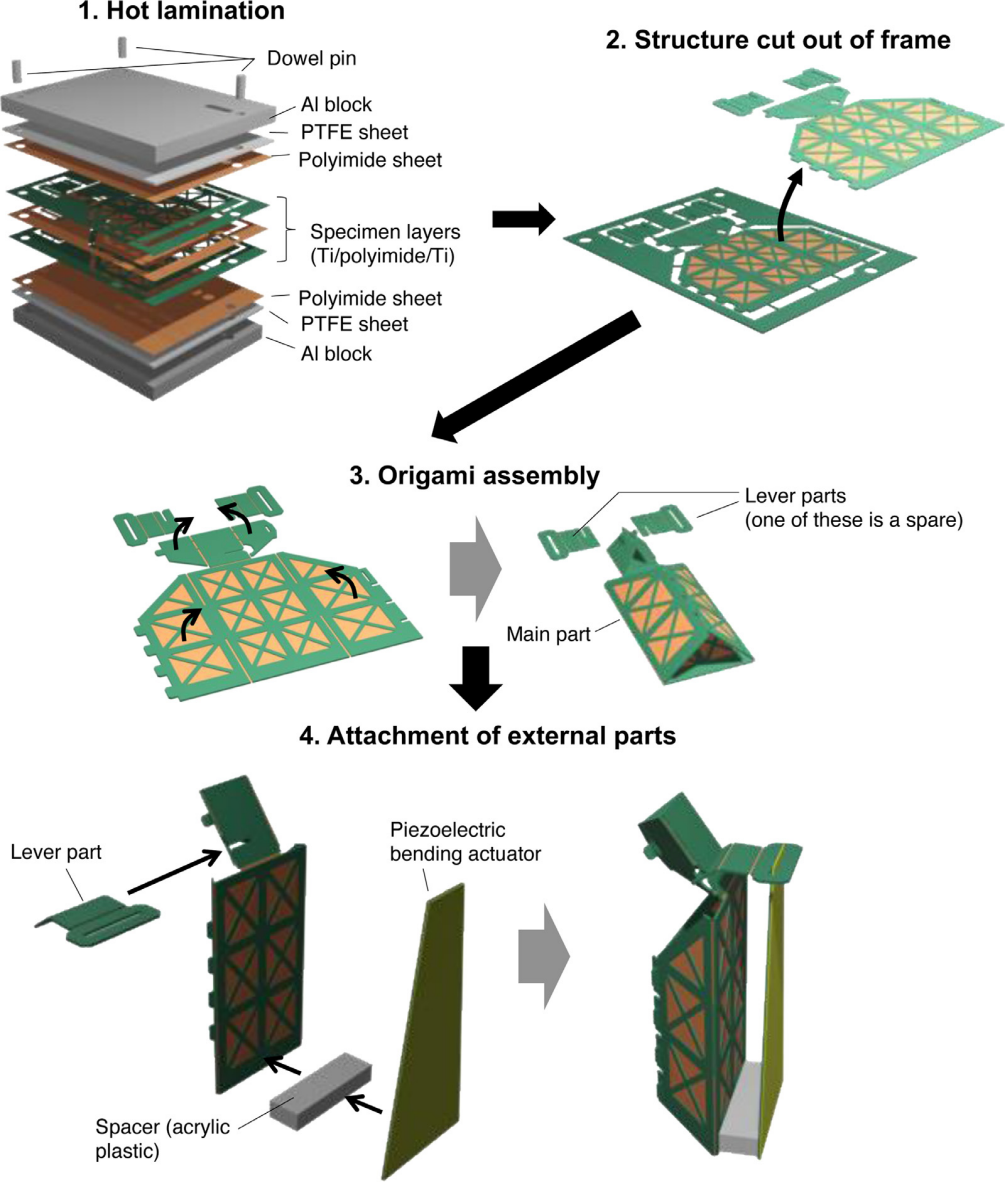
Fabrication process.

**Step 1)** The titanium/polyimide/titanium specimen layers were sandwiched between additional 50 µm-thick polyimide sheets (without thermal bondable layers), 1 mm-thick PTFE sheets, and aluminum blocks, as shown in Fig. 5. The PTFE sheets absorbed sample unevenness, and the polyimide sheets prevented the specimen layers from adhering to the PTFE sheets. We used UPILEX-S (Ube Industries, Ltd., Tokyo, Japan) and Nafron (NICHIAS Corporation, Tokyo, Japan) for the polyimide and PTFE sheets, respectively. Three alignment dowel pins were placed on the aluminum blocks. This stack was then heated and pressed at a temperature of 310 °C and a pressure of 0.5 MPa. We used typical pneumatic-actuated hot-pressing instruments (IMC-180C; Imoto Machinery Co., Ltd., Kyoto, Japan). The hot lamination lasted for 10 min. Alternatively, performing this hot-pressing with just a hot plate and weight is possible, which should lower the equipment investment Because the thermal expansion coefficients of titanium and polyimide are significantly different (8.4 and 16 ppm/K, respectively), the upper and lower titanium layers should have identical designs. Otherwise, the laminated specimen becomes largely warped.

**Step 2)** After the specimen was cooled, the structure was cut out of the outer frame using diagonal cutting pliers.

**Step 3)** The structure was assembled using an origami-like folding process. In our demonstrations, we reshaped some sections of the structure into prisms for increased rigidity. To fix the assembled part, we used a UV-curable adhesive (Loctite 4305; Henkel AG & Company, Düsseldorf, Germany) or epoxy adhesive (Quick 5; Konishi Co., Ltd., Osaka, Japan). UV adhesives can be cured using low-cost commercial UV lights.

**Step 4)** Finally, additional external parts can be attached to the fabricated mechanism depending on the application and purpose. In our study, we attached a spacer block (acrylic plastic) and a 26 mm-long piezoelectric bending actuator. The latter used in this experiment was composed of lamination of piezoelectric single crystalline Pb(In_1/2_Nb_1/2_)O_3_-Pb(Mg_1/3_Nb_2/3_)O_3_-PbTiO_3_ (PINPMN-PT) and an elastic titanium layer. The detailed shape and dimensions were the same as those of the actuator described in Ref. [10].

## Results and actuation demonstration

**Fig. 6:**
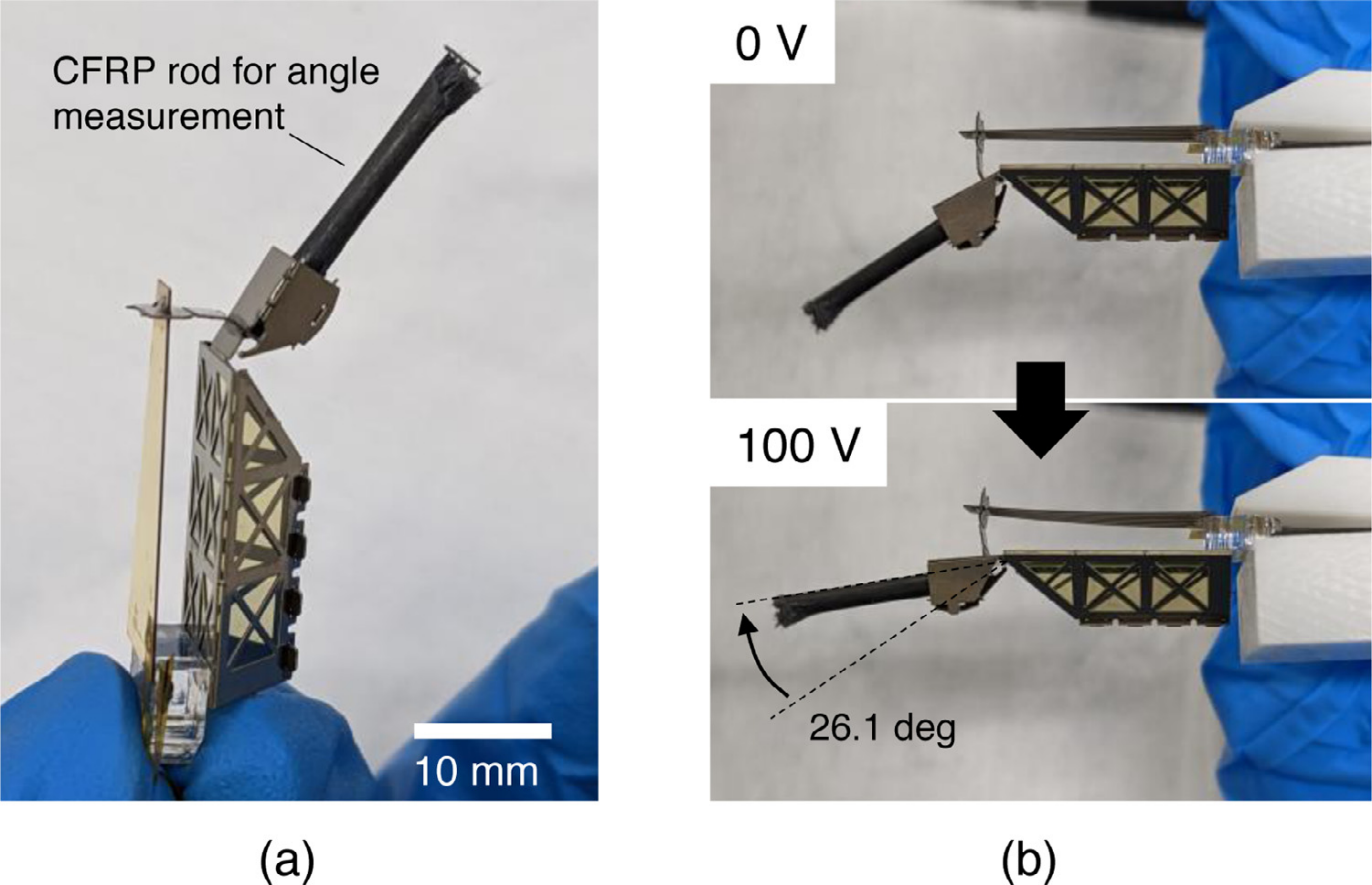
Fabricated demonstration mechanism. (a) Appearance and (b) actuation demonstration.

Fig. 6a shows a photograph of the fabricated demonstration mechanism. Here, a CFRP rod was attached to the tip of the rotating part to make it easier to observe the rotation angle. Fig. 6b shows the piezoelectric actuator under an applied voltage of 100 V. The displacement angle was 26.1°. The unloaded tip of the piezoelectric actuator was displaced by 0.95 mm when 100 V was applied. Thus, the ideal rotation angle was arcsin(0.95mm/2.0mm)= 28.4°. The experimental results had an error value of 8.1%, which suggests a good agreement with the ideal value. This means that the fabricated structural geometry agreed well with the design.

We compare the CFRP used in our previous work [8] with the metal used here. The former has advantages of a high elastic modulus and strength at low density (1500 kg/m^2^), and its density is three and five times lighter than titanium or stainless steel, respectively, making it suitable for applications where lightness is critical. In the study [8], CFRP is actually applied to a flying robot where lightness is the most important. On the contrary, as mentioned in the introduction, CFRP is disadvantageous in some practical productions, such as edge fraying during processing, low heat resistance, and high material cost. In our proposed process, these problems can be solved using a metal layer. The employment of a metal layer allows using the adhesive-less polyimides, which also contributes to reducing the number of layers required; the adhesive-less polyimide is not applicable to CFRP due to its high bonding temperature of 310 °C or higher. In summary, our proposed process is a more attractive option for applications where the structural weight is not a bottleneck. We also confirmed that this process can be realized with a minimum equipment cost of only a hot plate and weights and a low outsourcing cost (which varies from case to case, but here, the cost was about $1,000) through the experiments.

## Conclusion

We developed a new method for fabricating micro three-dimensional mechanisms and demonstrated it by fabricating a miniaturized transmission mechanism for a piezoelectric bending actuator. The polyimide hinge mechanism worked as designed, demonstrating the effectiveness of this method. The layers in our sample mechanism were 42 mm × 32 mm in size; however, a larger hot press could be used to make larger samples or multiple samples. In addition, hot pressing took only ~1 h, including heating and cooling time for the instrument we used. The subsequent steps can also be completed rapidly using UV-curing adhesives; therefore, we believe that this method has an excellent throughput. Our method could be implemented using only a hot plate (and weight) and external manufacturer(s) capable of metal-wet etching and polyimide laser cutting. We expect that these requirements will be easily met by many researchers, and our method will therefore be potentially useful to researchers developing microrobots.

## Declaration of Competing Interest

The authors declare that they have no known competing financial interests or personal relationships that could have influenced the work reported in this paper.

## Appendix Supplementary materials

Image, application 1
